# Effect of Calcination Temperature on the Physicochemical Properties and Electrochemical Performance of FeVO_4_ as an Anode for Lithium-Ion Batteries

**DOI:** 10.3390/ma16020565

**Published:** 2023-01-06

**Authors:** Faizan Ghani, Kunsik An, Dongjin Lee

**Affiliations:** 1 Department of Mechanical and Aerospace Engineering, Konkuk University, Seoul Campus, 120 Neungdong-ro, Gwangjin-gu, Seoul 05029, Republic of Korea; 2 Department of Mechatronics Engineering, Konkuk University, Glocal Campus, 268 Chungwon-daero, Chungju-si 27478, Republic of Korea

**Keywords:** anode, calcination temperature, electrochemical performance, FeVO_4_, hydrothermal, Li-ion battery, physicochemical properties

## Abstract

Several electrode materials have been developed to provide high energy density and a long calendar life at a low cost for lithium-ion batteries (LIBs). Iron (III) vanadate (FeVO_4_), a semiconductor material that follows insertion/extraction chemistry with a redox reaction and provides high theoretical capacity, is an auspicious choice of anode material for LIBs. The correlation is investigated between calcination temperatures, morphology, particle size, physicochemical properties, and their effect on the electrochemical performance of FeVO_4_ under different binders. The crystallite size, particle size, and tap density increase while the specific surface area (S_BET_) decreases upon increasing the calcination temperature (500 °C, 600 °C, and 700 °C). The specific capacities are reduced by increasing the calcination temperature and particle size. Furthermore, FeVO_4_ fabricated with different binders (35 wt.% PAA and 5 wt.% PVDF) and their electrochemical performance for LIBs was explored regarding the effectiveness of the PAA binder. FV500 (PAA and PVDF) initially delivered higher discharge/charge capacities of 1046.23/771.692 mAhg^−1^ and 1051.21/661.849 mAhg^−1^ compared to FV600 and FV700 at the current densities of 100 mAg^−1^, respectively. The intrinsic defects and presence of oxygen vacancy along with high surface area and smaller particle sizes efficiently enhanced the ionic and electronic conductivities and delivered high discharge/charge capacities for FeVO_4_ as an anode for LIBs.

## 1. Introduction

The search for new anode materials with low density, cost, and good electronic and ionic conductivity continued since the commercialization of lithium-ion batteries [1]. Graphite and Li_4_Ti_5_O_12_ (LTO), intercalation/de-intercalation materials providing a long cycle life with specific capacities of 378 mAhg^−1^ and 175 mAhg^−1^, justify the need for an anode material for commercial lithium-ion batteries [2,3]. These materials have problems with Li dendrites formation, internal short circuits, low theoretical specific capacities, and poor electronic conductivity of LTO, and research to overcome these challenges is still in progress [4,5]. Conversion materials are a better choice of anode material because of their high theoretical specific capacity (>600 mAhg^−1^), safety, environmental sociability, and low cost [6]. Transition metals attribute multiple oxidation states, form numerous oxides, and deliver a theoretical specific capacity of approximately 700 mAhg^−1^ with 100% capacity retention, proving to be an excellent choice of anode materials [7]. Vanadium is an abundant element of the earth’s crust with several oxidation states (+2, +3, +4, +5), coordination numbers (4–6), and Li^+^ ions intercalation/de-intercalation aptitude, which have branded it a suitable element for the electrode materials of LIBs [8]. Iron (III) vanadate (FeVO_4_), a binary oxide material following the mixed-reaction mechanism, is easy to synthesize and delivers a theoretical specific capacity of ~1255.5 mAhg^−1^ and can be a virtuous anode material for LIBs. Different synthesis conditions formulate different crystal structures of FeVO_4_ in the form of FeVO_4_-I, FeVO_4_-II, FeVO_4_-III, and FeVO_4_-IV simultaneously [9]. Among them, FeVO_4_-I contains VO_4_ tetrahedrons, which share their corners with O_2_, and two distorted FeO_6_ octahedra that share an edge with one distorted FeO_5_ trigonal bipyramid and produce a doubly bent structure. The distortion of polyhedrons between FeO_6_ and FeO_5_ increases due to the ionic repulsion of Fe and V present in an oxidation state of +3 and +5, respectively [10]. Diffuse reflectance spectral studies have predicted the lattice defects associated with oxygen deficiency and V^+4^ ion presence in FeVO_4_ [11]. FeVO_4_ is a confirmed n-type semiconductor whose conduction is thermally activated by the electron-hopping mechanism [12].

The quantum mechanical approach to nanostructured materials explains their different behavior compared to bulk materials, and their excessive electrical, mechanical, and transportation properties have overwhelmed modern research on catalysis, photoluminescence, gas sensing, solar cells, and energy storage devices [13]. Abundant synthesis approaches such as co-precipitation, sol-gel, and hydrothermal reaction have been used to prepare particles of different shapes and sizes. The synthesis methods of FeVO_4_ such as co-precipitation, sol-gel, microemulsions, etc., require careful control of several synthesis conditions such as the synthesis temperature, synthesis time, pH, effect of concentration, calcination temperature, calcination time, etc. These synthesis conditions increase the synthesis time and cost and demand considerable experimentation. Various surfactants and template materials can be used to achieve different morphologies [14,15,16]. Therefore, we acquired hydrothermal synthesis methods to avoid the addition of surfactants/template materials and pH control steps. The nanocrystalline FeVO_4_ was synthesized via the precipitation process to measure the photocatalytic activity of visible light [17]. FeVO_4_ nanorods were prepared by acquiring the hydrothermal methodology using different precursors for gas sensing activity [18,19]. It is reported that optimizing the calcination temperature can enhance the rate capability of triple-shelled ZnFe_2_O_4_ hollow microspheres [20]. The optimization of the calcination temperature determined that ZnFe_2_O_4_ calcined at 700℃ delivered high cyclic and rate performances as an anode for LIBs [21]. Furthermore, novel synthesis of the ZnO/C composite at different calcination temperatures changes the morphology, surface area, and associated electrochemical performance as an anode for LIBs [22]. Therefore, it is necessary to investigate the correlation of particle and physicochemical characteristics with different calcination temperatures, as well as their electrochemical performance.

Moreover, developing a metal–organic framework (MOF) can also strengthen the physicochemical and electrochemical properties of FeVO_4_ without regard for the synthesis time, cost, and addition of extra organic/polymeric materials [23,24]. FeVO_4_ synthesized by the ‘chimi douce’ method delivered a specific capacity of 1200 mAhg^−1^ for LIBs, which continued to decay upon further cycling [25]. Ce-doped FeVO_4_ nanoparticles exhibited better retention capacity, achieving 578 mAhg^−1^ as an anode material for LIBs for up to 40 cycles [26]. We previously reported a 60.53% retention capacity after 100 cycles at 0.1C for FeVO_4_ hollow microspheres used as an anode for LIBs [27]. N. Yan et al. showed a very stable cycle performance of FeVO_4_ [28]. Numerous spectroscopy techniques reported that the reaction mechanism of FeVO_4_ and its origin of extra capacity was associated with the redox coupling of Fe and V and anion activity (R-O/V-O) for LIBs. Electrochemical grinding of material during the cycling process creates a Li-O interaction to deliver the extra capacity of FeVO_4_ [29,30,31,32]. Moreover, the PVDF binder containing fluorine atoms with poor mechanical properties and elasticity generates LiF, HF, and PF_5_, disintegrates the electrode particles, and dissolves the electrolytes. The PAA binder, on the other hand, with its high viscosity, mechanical strength, and adequate adhesive properties, effectively withstands the volume changes during the charging process [33,34]. Therefore, an appropriate choice of a binder for the FeVO_4_ anode material should be investigated. The emphasis of our experimentation is on investigating how different calcination temperatures affect the particles, physicochemical properties, and electrochemical performance, as well as the effect of different binders on the electrochemical performance of FeVO_4_. Furthermore, we explore the correlation between the calcination temperature, particle distribution, and physicochemical characteristics, as well as the electrochemical performance. We use ammonium metavanadate and iron chloride hexahydrate as precursor materials, and follow the Ostwald ripening synthesis route to overcome the problem of controlling the pH during the synthesis of FeVO_4_.

## 2. Materials and Methods

All the reagents were of analytical grade and were used as received. Iron chloride hexahydrate (FeCl_3_·6H_2_O) was purchased from Kanto Chemical Industries Japan. Ammonium metavanadate (NH_4_VO_3_), the vanadium source, a 35-weight % PAA solution, and PVDF crystal powder were purchased from Sigma Aldrich. In a typical synthesis, 2 mmol of FeCl_3_·6H_2_O was dissolved in 10 mL of Di water, and another 2 mmol of NH_4_VO_3_ was dissolved in 10 mL of Di water at 90 °C_._ After dissolving the precursors, the NH_4_VO_3_ solution was added dropwise into the FeCl_3_·6H_2_O solution to initiate the double replacement reaction. After mixing for 30 min, the solution was transferred to a 50 mL Teflon-lined stainless-steel autoclave and heated at 180 °C for 3 h to complete the Ostwald ripening process. The naturally cooled, orange-colored precipitates were collected, washed several times with water and ethanol to remove impurities, vacuum dried at 60 °C overnight, and calcined at 500 °C, 600 °C, and 700 °C for 2 h at the heating rate of 5 °C/min. Finally, the calcined powders were denoted as FV500, FV600, and FV700, respectively, for further characterization.

A 35-weight % PAA solution without any further treatment was used for electrode fabrication. In contrast, a 5-weight % PVDF binder solution was prepared by mixing the stoichiometric amount of PVDF powder in 20 mL of the NMP (2-N-Methyl-pyrrolidone) solvent. The suspension solution was stirred at room temperature in a closed container for 24 h to achieve a transparent, homogeneous, and uniform PVDF binder solution. 

### 2.1. Material Characterizations

The phase purity and crystallinity of FV500, FV600, and FV700 were examined by powder X-ray diffraction (XRD; Rigaku D/MAX-2500 V/PC, Tokyo, Japan) furnished with Cu-Kα (λ = 1.54046 Å) radiation. The particle sizes and shapes were analyzed with the help of a field emission scanning electron microscope (FE-SEM; S-4200, Hitachi, High Technologies, Tokyo, Japan). The morphology and surface textures of FV500, FV600, and FV700 were further confirmed by energy-filtered transmission electron microscopy (EF-TEM, Titan, FEI, Corp., Hillsboro, OR, USA). The average particle size and particle size distributions of FV500, FV600, and FV700 were measured by ImageJ software. The N_2_ adsorption/desorption isotherms were recorded with the BJH N_2_ adsorption instrument (Belsorp II mini, BEL Japan, Inc., Tokyo, Japan). The effect of different calcination temperatures of FeVO_4_ was characterized using Raman spectroscopy (Renishaw Model at λ = 520 nm), and oxidation states/chemical compositions were studied with the X-ray photoelectron spectroscopy (PHI 5000 VersaProbe (Ulvac-PHI)) equipped with an Al Kα (1486.6 eV) Monochromator.

### 2.2. Electrochemical Characterizations

The electrochemical properties of FV500, FV600, and FV700 were measured by assembling a coin-type cell (CR2032). The working electrodes were prepared by uniformly and homogenously mixing the active material, acetylene black (to enhance the electronic conductivity), and binder material in a mixing ratio of 60:20:20. Two different binder materials [35 weight % polyacrylic acid (PAA) in ethanol solvent and 5 weight % of polyvinylidene fluoride (PVDF) in NMP (2-N-Methyl-pyrrolidone) solvent] were used for fabricating the working electrodes of FV500, FV600, and FV700. The active mass loading amounts and electrode thicknesses were maintained at ~0.91–1.3 mg/cm and ~26–32 μm for FV500, FV600, and FV700, respectively. The as-prepared electrodes were dried at 60 °C for 6 h and roll-pressed to optimize the electrodes’ thicknesses and compact densities. The as-prepared electrodes were cut into coin size, vacuum dried at 80 °C for 4 h, and assembled in an Argon-filled glove box (Glove Box System, KK–011–AS, South Kore) with Li foil as a counter electrode and 1.2 M LiPF_6_ in EC: DEC (1:1) (*v*/*v*) as the electrolyte and polypropylene separator. The cells were tested at the current density of 100 mAg^−1^ within the voltage range of 0.005 V–3.0 V at room temperature (25 °C) using galvanostat/potentiostat (Multi–Cycling Battery & Capacitor Test System, Series 4000, Korea Thermo–Tech Co., Ltd., Seoul, Republic of Korea). Cyclic voltammetry (CV) was analyzed using a Biologic potentiostat/galvanostat Model VMP3 (BioLab, Inc. Pariset, France) at 0.1 mVs^−1^ in a voltage window of 0.005 V–3.0 V.

## 3. Results

### 3.1. Synthesis Mechanism and Reaction Chemistry 

**Scheme 1:**
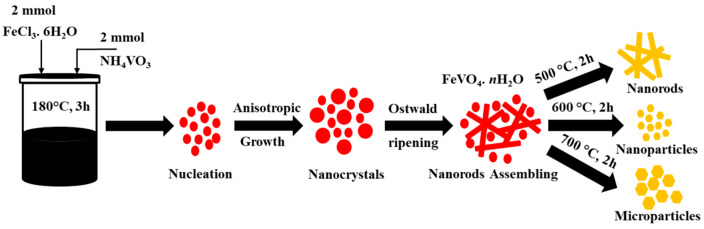
Systematic illustration of synthesis mechanism scheme of FeVO_4_.

The systematic synthesis illustration of Scheme 1 depicts the reaction chemistry of our synthesis mechanism in the following four steps: (i) Hydrolysis of the precursor materials: The precursor materials, NH_4_VO_3_ and FeCl_3_.6H_2_O, were initially hydrolyzed under specific stirring conditions to provide Fe^+3^ and VO^−3^ ions in an aqueous solvent. (ii) The disproportionation reaction proceeded following the hydrolysis step, generating metallic ligands ((a) a complex-metallic coordinated compound), which initiated the nucleation process. The nucleation process nucleated the anisotropic growth of inhomogeneous FeVO_4_ nanocrystals. (iii) The inhomogeneity of FeVO_4_ nanocrystals produced multiple activation energies, which followed the Ostwald ripening process. FeVO_4_ nanocrystals with lower activation energies elongated in a unidirectional way to take the shape of nanorods until achieving equilibrium. The Ostwald ripening process homogenously transformed all the FeVO_4_ nanocrystals into thermodynamically stabilized nanorods. The as-prepared nanorods (FeVO_4_ nH_2_O) with attached water molecules therefore required calcination at different temperatures to release water molecules and investigate particle properties. (iv) The calcination process at different temperatures (500 °C, 600 °C, and 700 °C) liberated water molecules and other impurities to grow the FeVO_4_ crystal structure and provided different morphologies and particle properties. The calcination of as-prepared nanorods (FeVO_4_ nH_2_O) released water molecules and deformed grain boundaries and crystallite sizes to produce porous nanorods, which spontaneously broke down into nanoparticles and microparticles under different calcination temperatures (500 °C, 600 °C, and 700 °C). Therefore, different calcination temperatures provide different morphologies, influence the physicochemical properties, and change the electronic and ionic diffusion properties of Li^+^ ions of FeVO_4_ as the anode material of LIBs.

### 3.2. Phase and Crystal Structure Investigation

**Figure 1:**
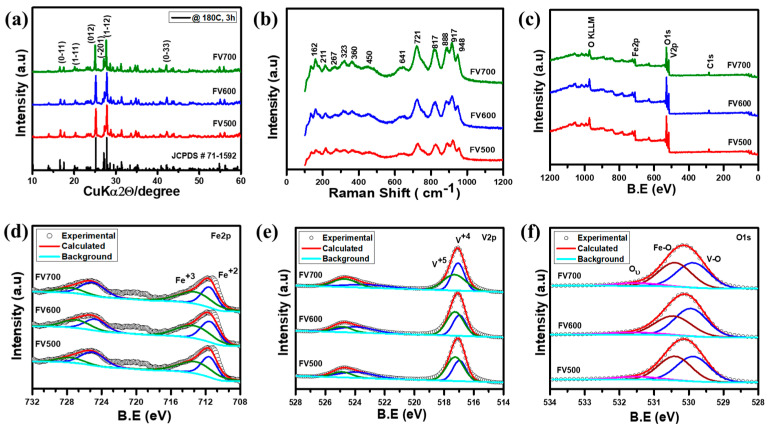
(**a**) XRD Analysis, (**b**) Raman spectroscopy analysis, (**c**) XPS survey spectrum, (**d**) XPS spectrum of Fe2p, (**e**) XPS spectrum of V2p, and (**f**) XPS spectrum of O1s of FV500, FV600, and FV700, respectively.

Figure 1a depicts the XRD patterns of FV500, FV600, and FV700, which aptly matched JCPDS Card #071-1592 of the triclinic phase of space group P-1(2). All the observed XRD peaks were highly crystalline in nature, with low peak intensities, and indexed with hkl parameters as (0–11), (1–11), (012), (−201), (1–12), (0–33), and (−412), respectively. The increase in calcination temperature increases crystallite size, deforms grain boundaries and internal strains, and enhances the intensities of XRD peaks. It is confirmed that the increase in the calcination temperature of FeVO_4_ (FV500, FV600, and FV700) directly affects the internal strains and increases their crystallite size, respectively (Table 1). Figure S1a exhibits the XRD pattern of as-synthesized FeVO_4_·nH_2_O along with the Rietveld refinement analysis of FV500, FV600, and FV700 shown in Figure S1b–d using FullProf Suit software. The unit cell parameters, lattice strains (ɛ), and crystal structures of FV500, FV600, and FV700 are summarized in Table 1. Rietveld refinement analysis indicates the perfect match between calculated and experimental XRD patterns and reveals the crystal structure of FeVO_4_-I. The crystal structure of FeVO_4_-I consists of VO_4_ tetrahedrons that share their corners with O_2_ atoms and two distorted FeO_6_ octahedrons that share the edge with one distorted FeO_5_ trigonal bipyramid to form a doubly bent structure. The distortion of the FeO_6_ and FeO_5_ polyhedrons primarily appeared because of the ionic repulsion of Fe atoms located between FeO_6_ and FeO_5_ polyhedrons. Furthermore, Raman spectroscopy identified the molecular structure of FV500, FV600, and FV700, and the corresponding results are shown in Figure 1b. Raman bands observed at the peak positions of 162, 211, and 267 cm^−1^ correspond to the stretching modes of vibration of Fe-O bonds. Raman bands at 323, 360, and 450 cm^−1^ determine the deformation of V-O-V bonds. Raman bands at 641, 721, and 817 cm^−1^ were associated with the stretching or mixed bridging modes of vibration of V-O-Fe bonds and stretching modes of vibration of V-O-Fe bonds, respectively. The terminal stretching vibration modes of V-O bonds were detected at 888, 917, and 948 cm^−1^, respectively. These Raman bands confirm the formation of the triclinic phase of FeVO_4_, which comprises VO_4_ tetrahedrons that share their corners with O_2_ and two distorted FeO_6_ octahedrons sharing its one edge with distorted FeO_5_ trigonal bipyramid to make a doubly bent structure. The distortion of the polyhedrons was observed because of the ionic repulsion of Fe between FeO_6_ and FeO_5_. Thus, XRD patterns and the Raman spectrum in Figure 1a,b confirm the same crystal structure of FeVO_4_ with a triclinic phase of space group P-1(2). The increased crystallite size and internal strain studied by the phonon distortion of Raman spectroscopy provide high-intensity peaks with an increasing calcination temperature. Therefore, the peak intensity of Raman bands of FV500, FV600, and FV700 increases with an increasing calcination temperature due to the enhanced degree of crystallization, as can be determined by the corresponding increase in the crystallite size of FeVO_4_ [35,36]. Therefore, the order of crystallinity is observed as follows: FV700 > FV600 > FV500. XPS analysis shown in Figure 1c–f exhibits the oxidation states, chemical composition, and crystal structures of FV500, FV600, and FV700, respectively. The survey spectrum of FV500, FV600, and FV700 shown in Figure 1c confirms the presence of Fe, V, and O elements without any other impurity element. The difference in binding energies of 13.9 eV between Fe2p_3/2_ and Fe2p_1/2_ orbitals of the Fe2p spectrum shown in Figure 1d corresponds to Fe^+3^ oxidation states. Fe2p_3/2_ and Fe2p_1/2_ orbitals indicate two deconvolution peaks associated with the oxidation states of Fe^+2^ and Fe^+3^ at the binding energies of 712.20/713.70 eVs and 725.80/727.57 eVs, respectively. Similarly, the difference in binding energies of 7.64 eV between V2p_3/2_ and V2p_1/2_ orbitals of the V2p spectrum shown in Figure 1e corresponds to V^+5^ oxidation states. V2p_3/2_ and V2p_1/2_ orbitals deconvoluted into two peaks at the binding energies of 516.97/517.26 eV and 523.92/524.88 eV, consistent with the oxidation states of V^+4^ and V^+5^, respectively. The O1s spectrum displayed in Figure 1f deconvoluted into three peaks at the binding energies of 529.87 eV, 530.40 eV, and 531.50 eV analogous to V-O, Fe-O (metal-oxygen bonds), and O_υ_ (oxygen vacancies), for FV500, FV600, and FV700, respectively. The presence of O_υ_ (oxygen vacancies) and V^+4^ along with Fe^+2^/Fe^+3^ (cationic defects) confirms the electron-hopping mechanism to enhance the electronic and ionic conductivities of FV500, FV600, and FV700 [11,12]. The presence of O_υ_ (oxygen vacancies) and cationic defects transforms FeVO_4_ into a semiconductor material and hypothetically proves it to be an auspicious anode material for LIBs. 

### 3.3. Physicochemical Properties and Particles Characteristics

**Figure 2:**
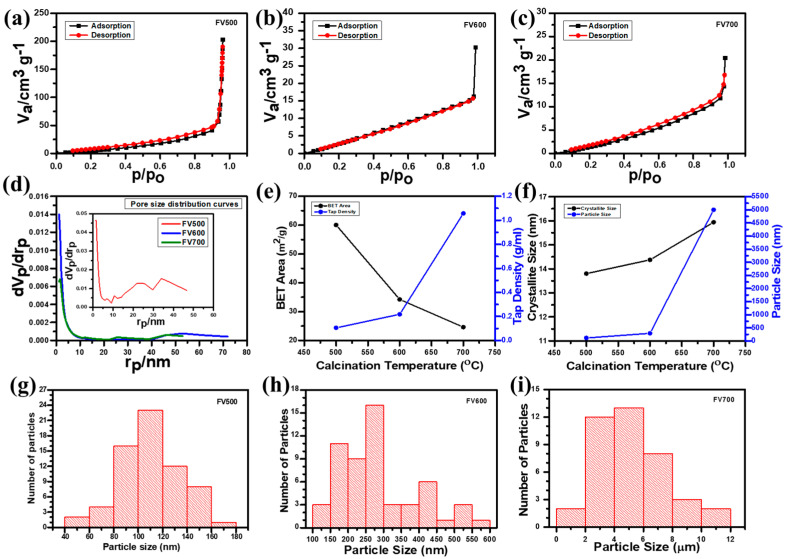
N_2_ adsorption/desorption curves of (**a**) FV500, (**b**) FV600, and (**c**) FV700, respectively. (**d**) pore size distribution curves of FV500, FV600, and FV700. The correlation between (**e**) crystallite size, particle size, and (**f**) BET area, and tap density at different calcination temperatures (FV500, FV600, and FV700). The particle size distribution curves calculated by ImageJ software of (**g**) FV500, (**h**) FV600, and (**i**) FV700, respectively.

N_2_ adsorption/desorption isotherms demonstrated in Figure 2a–c are obtained after degassing the FV500, FV600, and FV700 at 120 ℃ for 6 h. The same reaction chemistry and mechanism provide the same kind of isotherms. The different calcination temperatures and particle sizes produced a slight change in the shape of type III BET isotherms. Type III BET isotherms verify the existence of weak interactions between N_2_ gas molecules (adsorbate) and FV500, FV600, and FV700 (adsorbents). The low heat of adsorption (c < 1) leads to a weak Van der Waals interaction between the adsorbate and adsorbent and confirms multilayer formation at low to medium pressures. Furthermore, type III isotherms did not show the formation of an asymptote at the medium pressure range, verifying the multilayer formation with a non-porous nature. The multilayer adsorption undergoes a capillary condensation process near the supersaturation pressure (P/P⁰ = 1). Thus, N_2_ adsorption/desorption isotherms with a missing monolayer exhibit a nonporous nature of FV500, FV600, and FV700. Figure 2d demonstrates the pore size distribution curves with mesopores and macropores for FV500, FV600, and FV700, respectively. The observed mesopores and macropores for FV500, FV600, and FV700 were the result of intraparticle interactions (voids) rather than interparticle interactions. The specific surface area (S_BET_), pore size, and pore volume were measured using the Brunauer–Emmett–Teller (BET) and Barrett–Joyner–Halenda (BJH) methods and are summarized in Table 2 along with the tap densities of FV500, FV600, and FV700. The S_BET_, pore sizes, and tap densities determined the surface-active sites, Li^+^ ions diffusion kinetics, and penetration of the electrolyte within the electrodes to affect the electrochemical performance of FV500, FV600, and FV700 as anode materials for LIBs. Figure 2e,f describe the effect of the calcination temperature on the physicochemical and particle characteristics. The specific surface area (S_BET_) continues to be reduced by increasing the calcination temperature from 500 °C to 700 °C as shown in Figure 2e. The reduction of the specific surface area (S_BET_) of FV500, FV600, and FV700 depends on the particle sizes and shapes as predicted in Figure 2f. Furthermore, the increasing trend of calcination temperature has a direct relationship with the tap densities of FV500, FV600, and FV700. Thus, it is concluded from Figure 2e that increasing the calcination temperature of FeVO_4_ can accommodate more active mass per unit volume of the electrode, which can enhance the specific capacity and energy density of LIBs. It is also concluded that decreasing the calcination temperature can enhance the specific surface area (S_BET_) of FeVO_4_, which describes the penetration of a large amount of the electrolyte to enhance the rate performance of the LIBs. Furthermore, Figure 2f shows that increasing the calcination temperature from 500 °C to 700 °C increases the crystallite size and enhances the particle size from nm to µm. It shows that particles constitute multiple crystallites and have a direct correlation with the calcination temperatures. Therefore, it is confirmed in Figure 2e,f that increasing the calcination temperature can directly affect the tap density and particle size and inversely influence the specific surface area (S_BET_), which can enhance the specific capacity, energy density, and rate performance of FeVO_4_ as an anode material for LIBs. Furthermore, the particle size distribution curves of FV500, FV600, and FV700 are also shown in the Figure 2g–i, depicting the average particle sizes of 115 nm, 275 nm, and 5.0 µm with a broad size distribution (FV700) and narrow size distribution curve (FV500 and FV600), respectively. FeVO_4_ with lower particle sizes deliver lower polarization and exhibited better capacity retention. Moreover, the narrow size distribution with smaller size promotes a better performance (specific capacity, cycle life, energy density, etc.,), and the broad size distribution consisting of microparticles/coarse particles promote lithium plating and threatens the thermal safety of battery operation, which decrease the performance of LIBs [37]. Therefore, we suppose that FV500 with a particle size of 115 nm can deliver high specific capacity, cycle life, and energy density as an anode material for LIBs compared to that of FV600 and FV700, having broad size distributions.

### 3.4. Morphology and Topology Characteristics

**Figure 3:**
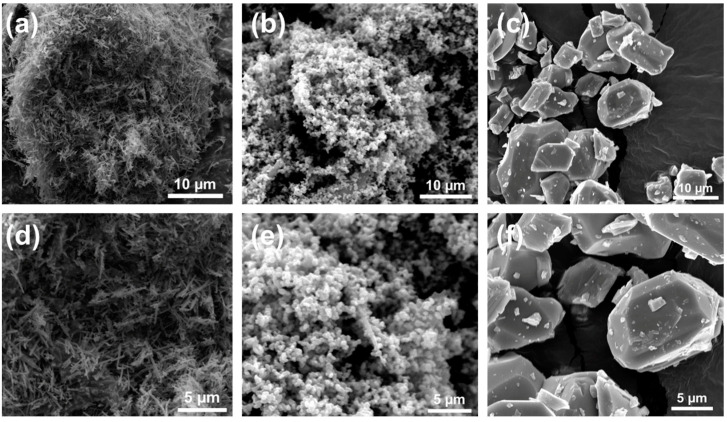
FE-SEM images of (**a**,**d**) FV500, (**b**,**e**) FV600, and (**c**,**f**) FV700 at the magnifications of 10 µm and 5 µm, respectively.

**Figure 4:**
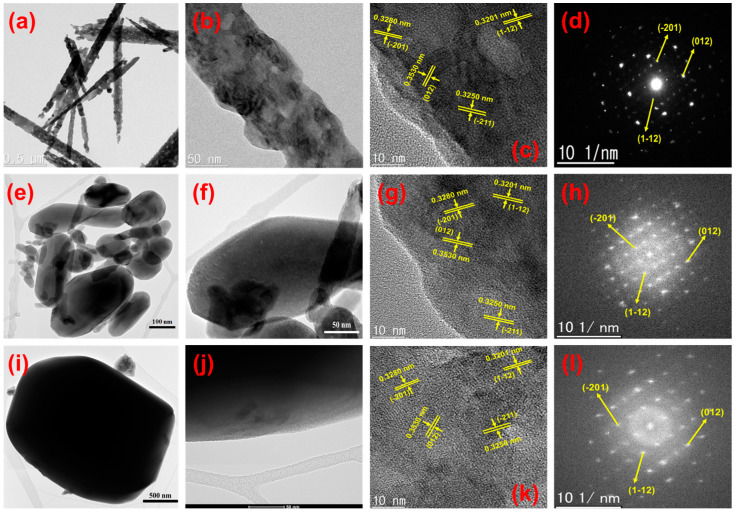
TEM images of (**a**,**b**) FV500, (**e**,**f**) FV600, and (**i**,**j**) FV700 and HRTEM images of (**c**) FV500, (**g**) FV600, and (**k**) FV700, respectively. SAED patterns of (**d**) FV500, (**h**) FV600, and (**l**) FV700, respectively.

SEM images shown in Figure 3 depict the morphology of FeVO_4_ after different calcination temperatures at various resolutions. The SEM images shown in Figure S2a,b exhibit the morphology of nanorods with different sizes and lengths of as-synthesized FeVO_4_. nH_2_O before calcination confirming the Ostwald ripening process during the hydrothermal reaction. Figure 3a,d show the morphology of nanorods with porous characteristics due to the release of water molecules from as-synthesized nanorods (FeVO_4_·nH_2_O) at the calcination of 500 °C (FV500). Figure 3b,e exhibit the aggregated morphology of nanoparticles of different sizes under the calcination temperature of 600 °C, which spontaneously break down porous nanorods into non-uniform, non-homogeneous polygonal nanoparticles (FV600). Furthermore, increasing the calcination temperature to 700 °C (FV700) homogenously produces multi-face polygonal microparticles as shown in Figure 3e,f. The calcination process under different temperatures (500 °C, 600 °C, and 700 °C) transforms the porous nanorods into polygonal nanoparticles and microparticles and deforms the grain boundaries and crystallite sizes. The transformation of porous nanorods into polygonal microparticles is associated with the large length-to-width ratio of FeVO_4_·nH_2_O nanorods. The average particle size of FV500, FV600, and FV70 was measured by ImageJ software and estimated as 115 nm, 290 nm, and 5.0 µm, respectively. Furthermore, the impedance of the smaller particles over large particles detected in Figure 3 confirms the Ostwald ripening process. The agglomeration of the multi-faceted hexagonal microparticles enhances the tap density and particle interaction during lithium insertion/extraction in the electrode. The particle’s size and shape moderate the Li^+^ ion diffusion during the cycling process of LIBs. The reduced particle size shortens the Li^+^ ions transportation length, which increases the Li^+^ ion diffusion and improves the cycle stability. In addition, Figure 4 exhibits TEM and HRTEM images probing the surface texture and elemental compositions of FV500, FV600, and FV700, respectively. The TEM images of Figure 4a,b clearly show the morphology of porous nanorods with the impedance of nanoparticles confirming the Ostwald ripening process at the calcination temperature of 500 °C. Similarly, TEM images of Figure 4e,f,i,j demonstrate the morphologies of polygonal nanoparticles and microparticles of FV600 and FV700, respectively. HRTEM images of Figure 4c,g,k reveal the interlayer d-spacings of 0.353 nm, 0.328 nm, 0.325 nm, and 0.320 nm, which corresponds to the hkl parameters of (012), (−201), (−211), and (1–12) of FV500, FV600, and FV700 of the XRD patterns of Figure 1a. Furthermore, SAED patterns of FV500, FV600, and FV700 illustrated in Figure 4d,h,l exhibit their corresponding polycrystalline nature, which agrees well with the XRD patterns of Figure 1a. Moreover, EDS mapping images of FV500, FV600, and FV700 shown in Figure S2 exhibit a similar elemental composition map for Fe and V resembling the atomic ratios of 1:1.2 for FV500, FV600, and FV700, respectively. The atomic ratio of FeVO_4_ measured from EDS mapping (Figure S2) is consistent with the atomic ratios of XPS analysis [Figure 1c–f]. The average particle size of FV500, FV600, and FV700 measured by ImageJ software was 2.35 μm × 115 nm (length × width), 287 nm, and 5.02 μm, respectively, and was associated with the average particle size measured by SEM images. Therefore, a self-assisted, self-controlled hydrothermal method under different temperatures of calcination (500 °C, 600 °C, 700 °C) can produce different morphologies with different particle sizes (nanorods, nanoparticles, and microparticles) of a pure crystalline phase of FeVO_4_-I.

### 3.5. Electrochemical Performance of FeVO_4_

**Figure 5:**
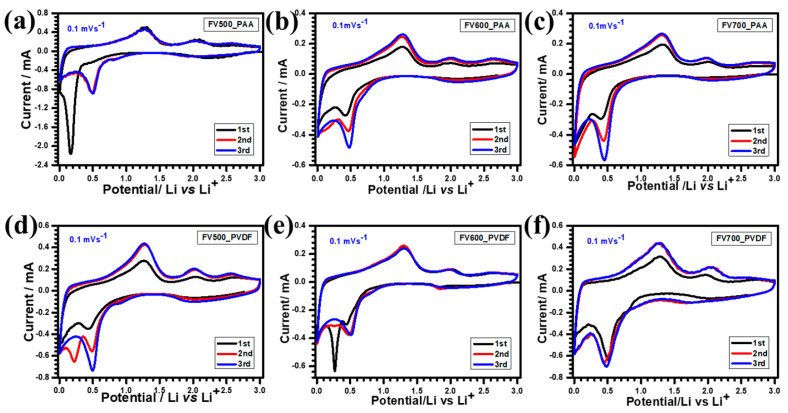
Cyclic voltammetry (CV) curves of (**a**–**c**) FV500, FV600, and FV700 electrodes fabricated with 35-weight % PAA and (**d**–**f**) FV500, FV600, and FV700 electrodes fabricated with 5-weight % PVDF binders at a scan rate of 0.1 mVs^−1^ within the voltage window of 0.005 V–3.0 V, respectively.

Electrochemical characterization for FV500, FV600, and FV700 was performed within the voltage range of 0.005 V–3.0 V and at the current density of 100 mAg^−1^ up to 50 cycles. Figure 5 displayed the CV curves of FV500, FV600, and FV700 with different binders (35 wt.% PAA and 5 wt.% PVDF) within the voltage range of 3.0 V–0.005 V and at the scan rate of 0.1 mVs^−1^, respectively. Figure 5a–c indicated the CV curves of FV500, FV600, and FV700 fabricated with the 35 wt.% PAA binder in ethanol solvent. The first cathodic scans of FV500, FV600, and FV700 electrodes exhibited reduction peaks at approximately 0.89 V, 0.5 V, and a sharp reduction peak at 0.2 V associated with the SEI layer formation and the conversion of FeVO_4_ into Li_x_V_2_O_5_ and metallic Fe^°^. The initial CV scan of FV600_PAA and FV700_PAA exhibited a reduction peak at 0.5 V similar to FV500_PAA. The anodic scan of the first cycle of FV500_PAA, FV600_PAA, and FV700_PAA indicated oxidation peaks at 1.27 V, 2.0 V, and 2.6 V associated with the conversion of Fe^°^/Fe^+3^, V^+3^/V^+4^, and V^+4^/V^+5^, respectively. The overlapping of the subsequent cycles with the same redox peaks confirmed a similar electrochemical reaction mechanism with the structural integrity of FeVO_4_ as _an_ anode for LIBs. FV600_PAA and FV700_PAA, with different particle sizes, tap densities, and morphologies, required a large number of currents in the following cycles for the insertion/extraction of Li^+^ ions. However, the initial CV scan of FV500_PVDF, FV600_PVDF, and FV700_PVDF exhibited a wide reduction peak at approximately 2.0 V, 0.9 V, and 0.5 V associated with the conversion reaction, SEI layer formation, and lithiation of Li_x_V_2_O_5_, respectively. An extra-sharp reduction peak at approximately 0.25 V appeared in the initial cathodic scan of FV600_PVDF and the second cycle of FV500_PVDF due to the metallic Fe° and polymeric gel formation. The anodic scan of FV500_PVDF, FV600_PVDF, and FV700_PVDF exhibited oxidation peaks at 1.27 V, 2.0 V, and 2.6 V associated with the conversion of Fe^°^/Fe^+3^, V^+3^/V^+4^, and V^+4^/V^+5^, respectively. The redox peaks overlapped in the following cycles, confirming a similar electrochemical reaction mechanism and structural integrity of FeVO_4_ as an anode for LIBs. Therefore, it is confirmed from the redox peaks of the CV curves that FeVO_4_ first undergoes lithium adsorption at the surface and is then converted into Li_x_V_2_O_5_ and Fe^°^ with the formation of the SEI layer. Li_x_V_2_O_5_ and Fe^°^ were transformed back into FeVO_4_ during the oxidation (charging) process. The electrochemical reaction mechanism of FeVO_4_ can be explained as:

FeVO_4_ + xLi^+^ + xe^−^ → Fe° + Li_x_V_2_O_5_ + Li_2_O (1)

**Figure 6:**
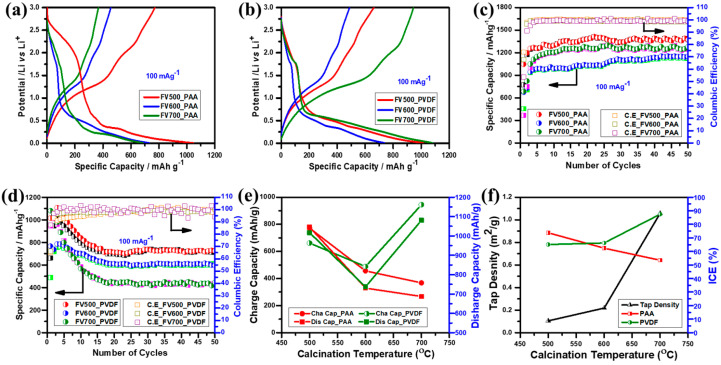
(**a**,**b**) Galvanostatic discharge/charge profiles and (**c**,**d**) cyclic performance of FV500, FV600, and FV700 at 35-weight % PAA and 5-weight % PVDF binders at current densities of 100 mAg^−1^ under the voltage range of 0.005 V-3.0 V, respectively (Color illustration: Black, Green, and Magenta colors represented the charge capacities; Red, Blue, and Olive colors represented the discharge capacities, respectively.). Correlation of (**e**) charge/discharge capacities and (**f**) tap density and initial coulombic efficiency of FV500, FV600, and FV700 at 35-weight % PAA and 5-weight % PVDF binders with different calcination temperatures, respectively.

The well-defined redox peaks observed for FV500_PAA electrodes due to the ion/electron transportation mechanisms demonstrated good reversibility of the working electrode. The current density of the redox processes increased significantly in the successive CV cycles due to the inhomogeneous particle sizes and morphologies of FV500, FV600, and FV700 electrodes for both binders (PAA and PVDF). Furthermore, the nature of the redox peaks observed in the following CV cycles of FV500, FV600, and FV700 electrodes for both binders (PAA and PVDF) indicated the analogous electrochemical reaction mechanism of FeVO_4_. Moreover, the overlapping of the subsequent CV cycles of FV500, FV600, and FV700 electrodes (PAA and PVDF) provides structural integrity and cycle stability during the cycling process. Therefore, it is concluded that (i) FV500, FV600, and FV700 follow the same electrochemical reaction mechanism, irrespective of the choice of binder. (ii) Physicochemical properties and morphology play an active role in influencing the electrochemical performance of FV500, FV600, and FV700, irrespective of binder selection. (iii) The 5-weight % PVDF binder can accommodate more thermodynamic changes during the cycling process because of its excellent cohesion/adhesion properties, but the presence of fluorine influences the dissolution of electrolyte to generate corrosive materials (HF, LiF, and PF_5_), which enhanced the current density of the redox processes and significantly affects the capacity fading for FeVO_4_. Therefore, 35-weight % PAA is a better choice of binder material for FV500, FV600, and FV700 with no fluorinated and toxicity of the PAA and solvent, superb adhesion/cohesion properties, and thermodynamic and mechanical stability, which influence the electrochemical performance of FeVO_4_ as an anode material for LIBs. Correspondingly, Figure 6a,b exhibit the first galvanostatic discharge/charge curves of FV500, FV600, and FV700 electrodes fabricated with different binders (35 wt.% PAA and 5 wt.% PVDF) within the voltage range of 0.005 V-3.0 V and at the current density of 100 mAg^−1^ for 50 cycles, respectively. FV500, FV600, and FV700 electrodes fabricated with a 35 wt.% PAA binder shown in Figure 6a delivered the discharge/charge capacities of 1046.23/771.69 mAhg^−1^, 730.70/455.99 mAhg^−1^, and 686.85/367.53 mAhg^−1^, respectively. Similarly, FV500, FV600, and FV700 electrodes fabricated with a 5 wt.% PVDF binder as shown in Figure 6b delivered the initial discharge/charge capacities of 1016.62/661.84 mAhg^−1^, 737.40/488.45 mAhg^−1^, and 1080.63/945.17 mAhg^−1^, respectively. The binder-free FeVO_4_/MXene electrode generated a synergistic effect to deliver the reversible capacity of 1179/1125 mAhg^−1^ at the current density of 100 mAg^−1^ after 250 cycles [38]. The FeVO_4_/graphene oxide composite delivered a reversible discharge capacity of 1046.5 mAhg^−1^ after 100 cycles at the current density of 100 mAg^−1^ [39]. FeVO_4_ hollow microspheres delivered the initial discharge capacity of 1378.82 mAhg^−1^ at the C-rate of 0.1C [27]. It is observed that electrodes fabricated with a PAA binder delivered higher initial coulombic efficiencies than that of PVDF binders except for the FV700_PVDF electrode. It is also observed that the FV700_PVDF electrode decreased below 0.1 V and delivered extra capacity because of the electrolyte and polymeric gel (LiCO_3_, HF, PF_6_, HCO_3_^−1^, etc.) decomposition during the discharging process. The discharge/charge curves of Figure 6a,b indicate the same voltage plateaus at different redox potentials for FV500, FV600, and FV700 electrodes (PAA and PVDF binders) as observed in the corresponding CV curves of Figure 5, confirming the similar electrochemical reaction mechanism. The discharge curves of electrodes fabricated with the PAA binder shown in Figure 6a indicated the high discharge capacities at lower voltages because of the conversion reactions compared to that of PVDF mixed electrodes. However, the initial discharge curves of electrodes fabricated with the PVDF binder shown in Figure 6b exhibited a larger number of Li^+^ ions inserted at the surface of FV500, FV600, and FV700 than that of PAA binder mixed electrodes. Moreover, Figure S4 indicates the first four discharge/charge curves of FV500, FV600, and FV700 electrodes (PAA and PVDF binders), respectively. It can be observed that the charge capacities of FV500, FV600, and FV700 electrodes (PAA and PVDF binders) increase after the initial cycle primarily due to the activation of local structural changes and the dissolution of electrolytes into HF, LiF, PF_6_, etc., respectively. These local structural changes under the thermal response of the electron-hopping mechanism of FeVO_4_ further enhance the electronic conductivity and Li^+^ ion diffusivity, which improves the charge capacities for FV500, FV600, and FV700 electrodes (PAA and PVDF binders) in the subsequent cycles. It is verified in previous reports that the PVDF binder with weak van der Waals forces and hydrogen bonding dissolved in electrolytes and released LiF, HF, and PF_5_-like acidic species that lead to a further volume expansion during the charging process and restricted the specific capacities. PAA is an appropriate choice of a binder for conversion and alloy/de-alloy-type electrodes because of their high adhesive properties, mechanical properties, strong hydrogen bonding with nonfluorinated species, and ability to accommodate large volume changes during the charging process [33,34,40]. 

Figure 6c,d indicate the cycling performance of FV500, FV600, and FV700 electrodes (PAA and PVDF binders) at the current density of 100 mAg^−1^ for 50 cycles, respectively. FV500, FV600, and FV700 electrodes fabricated with the PAA binder, as demonstrated in Figure 6c, deliver the initial coulombic efficiencies of 73.75%, 62.40%, and 53.50%, respectively. However, FV500, FV600, and FV700 electrodes fabricated with the PVDF binder, as shown in Figure 6d, exhibit initial coulombic efficiencies of 65.10%, 66.23%, and 87.46%, respectively. The coulombic efficiencies approached 98–99% after 50 cycles for FV500, FV600, and FV700 electrodes (PAA and PVDF binders), respectively. The capacity retention of FV500, FV600, and FV700 electrodes for PAA and PVDF binders were calculated as 131.33%, 157.70%, 186.20%, 71.45%, 82.11%, and 38.80%, respectively. Figure 6c,d demonstrate that the FV500 (nanorods) delivered higher specific capacity than FV600 and FV700 (polygonal nanoparticles and microparticles), respectively. Furthermore, the electrodes fabricated with the PAA binder exhibit higher specific capacities and cycle stability than that of PVDF binders. FV700_PAA delivers extra capacity at a lower voltage (<0.1 V) as a result of electrolyte dissolution and polymeric gel (LiCO_3_, HF, PF_6_, HCO_3_^−1^, etc.) decomposition during the discharging process. Figure 6d indicates that the PVDF binder electrodes exhibit a large capacity decrease in the initial 15 cycles because of the (i) unstable solid–electrolyte interface (SEI) layer formation, (ii) electrolyte decomposition, and (iii) Li^+^-ion consumption due to PVDF binder reactivity, respectively [27]. The instability of the SEI layer for PVDF binders was due to the numerous conversion reactions of Li_x_V_2_O_5_ at a lower voltage along with electrolyte decompositions, as shown below:

LiPF_6_ → LiF + PF_5_ (2)

H_2_O + PF_5_ → POF_3_ + 2HF (3)

The generation of corrosive HF during electrolyte decomposition intensifies the corrosion of active materials and promotes the capacity-fading mechanism. Although the instability of the SEI layer and electrolyte decomposition played a great role in the capacity-decaying process for both PAA and PVDF binder-fabricated electrodes, the degradation of binder particles further decreases the capacity-fading mechanism. The PVDF binder consumed even more Li^+^-ions during its degradation and generated HF and LiF compounds. The extra amount of HF and LiF irrespective of electrolyte decomposition caused the conductive contact of carbon to deteriorate and the specific capacity to decay [37,40,41]. It is verified in Figure 6c,d that the nanorods and nanoparticles deliver high specific capacities and cycle stability compared to that of FV700 electrodes. Furthermore, it is concluded that the PVDF binder suffers from the generation of HF, LiF, and PF_5_, which generate heat and lead to the decrease in capacity for FV500, FV600, and FV700 as anode materials for LIBs. In contrast, the PAA binder can accommodate more Li^+^ ions and decrease heat generation to deliver high specific capacity and cycle stability. The cycle stability trend can be explained as FV500 > FV600 > FV700, and the high specific capacities were the result of their morphology and smaller sizes of the particles. The morphology of nanorods provides large surface-active sites and formulated a large contact area between electrode particles, electrode-electrolytes, and electrode-current collectors, alleviated the lattice strains, and shortened the transportation length of Li^+^-ions during the cycling process. Figure 6e,f describe the effect of calcination temperature and tap density on the electrochemical performance of FV500, FV600, and FV700 as anode materials for LIBs. The tap density increases with the increase in the calcination temperature, which directly affects the discharge/charge capacities (PAA and PVDF) and coulombic efficiencies of FV500, FV600, and FV700. The discharge/charge capacities and coulombic efficiency of the FV700_PVDF electrode vary from the trend because of its electroactive activation at a lower voltage (<0.1 V) as a result of electrolyte dissolution and polymeric gel (LiCO_3_, HF, PF_6_, HCO_3_^−1^, etc.) decomposition during the discharging process. Therefore, it is concluded that the calcination temperature is a critical parameter that influences the physicochemical characteristics and electrochemical performance of FV500, FV600, and FV700 as anode material for LIBs.

## 4. Discussion

The electrochemical performance of electrode materials is primarily a function of materials’ crystal structure, particle size, shapes, and electronic conductivity to proliferate the diffusivity of Li^+^ ion intercalation/de-intercalation [42,43,44,45]. Quantum mechanics reveal that electrons have quantum confinement and unrestricted motions in different energy states of the orbital of an atom. In 2D material, nanoparticles, and microparticles, electrons are confined in the thickness direction, have certain unrestricted motions, and control the electrical conductivity. Particles with smaller grain boundaries produce more scattering, hence lowering the electrical conductivity [13,14,15,16,17,18,19,20,21,22,23,24,25,26,27,28,29,30,31,32,33,34,35,36,37,38,39,40,41,42,43,44,45,46]. The systematic synthesis scheme of FV500, FV600, and FV700 portrayed in Scheme 1 illustrates the mixing of equal amounts of Iron chloride hexahydrate and ammonium metavanadate under continuous stirring at 90 °C to nucleate the nanocrystal precipitates. The produced nanocrystals are of various sizes and have different amounts of energy at the surface and in the interior of the particles. This thermodynamic alteration of energy creates energetically favorable molecules (microparticles), which accumulate energetically unfavorable molecules (nanoparticles) over them. The shrinkage of unfavorable energy molecules and the extension of energetically favorable molecules produce large spherical particles. The elongation of these particles produces nanorods to achieve thermodynamic equilibrium, which, upon calcination at different temperatures, harvest nanoparticles and microparticles. The calcination temperature affects the particle nature, physicochemical characteristics, and electrochemical performance of FV500, FV600, and FV700. The size of the particle does matter. The number of surface atoms increases for nanoparticles via several dangling chemical bonds at the surface and, hence, increases the surface chemical reactivity [42]. As a result, the diffusion of Li^+^ ions enhances ion transportation and electrochemical performance. Moreover, the BET theory aptly explained the behavior of various particle sizes of FV500, FV600, and FV700. Particles with smaller sizes correspond to a higher specific surface area due to the reduced activation energy at the surface and enhanced reactivity as a result of the increased number of dangling bonds at the surface. The nanorods show higher specific capacities in contrast to nanoparticles and microparticles due to their size difference and surface area. FV500, FV600, and FV700 (PAA/PVDF) experimentally intercalated 6.66/6.69, 4.29/4.88, and 4.37/7.69 moles of lithium in the first discharge cycle, respectively. The number of intercalated lithium ions increased in the succeeding cycles and enhanced the specific capacities. We already knew that FeVO_4_ followed the electron-hopping mechanism, which shortens the energy bandgap between the valance and conduction bands at a specific temperature and acts as a semiconductor material. This hopping of electrons surges the electrical conductivity of the FeVO_4_ and highly accelerates the electron movements within the active material and leads to higher diffusivity of the Li^+^ ions. Moreover, the Li-O interaction that occurs during the electrochemical grinding and the intrinsic defects and oxygen vacancies in the FeVO_4_ crystal structure lead to higher specific capacity values. The increased specific capacities in the following cycles were the result of (i) re-structuring of the electrode material, (ii) the morphology and crystallinity of FV500, FV600, and FV700, which enhanced the tap density and surface area influencing the electrochemical performance of FeVO_4_ as anode for LIBs, (iii) the SEI layer and polymeric gel formation during the insertion/extraction of Li^+^ ions, (iv) and the active role of anion presence (oxygen redox-active center to form Li-O-Fe, and Li-O-V) during the cycling process. (v) The increased current density in the following redox processes, as mentioned in the CV analysis of Figure 5, enhanced the insertion/extraction of Li^+^ ions. Therefore, it can be concluded that physicochemical properties and the electrode structure can effectively enhance the electrochemical performance of FeVO_4_ as an anode material for LIBs, the SEI layer and polymeric-gel formation, the presence of anions (oxygen redox-active center to form Li-O-Fe, and Li-O-V), morphologies, particle size, and large surface areas, respectively [32]. There exist large activation sites over the surface of FV500 and FV600 in contrast to FV700 due to the increased number of dangling bonds, which decreases the thermodynamic parameters, such as surface tension, melting point, and heat capacity, hence promoting highly reactive surfaces. The Li^+^ ions occupy the surface of FV500 and FV600, leading to superior diffusion kinetics and improving their electrochemical performance as anode materials for LIBs. In addition, the large volume expansion causes a large capacity decay, which occurred due to the occurrence of irreversible phase transitions, the collapsing of the electrode structure, degradation of the binder particles and dissolution of binders in electrolytes, and the generation of acidic species such as HF, LiF, and PF_5_ during the cycling process [47,48]. Therefore, FV500 and FV600 provide high specific capacities because of their particle sizes, morphology, and surface area as compared to FV700 and confirm FeVO_4_ as an auspicious anode material for LIBs.

## 5. Conclusions

Different particle size distributions, morphologies, and physicochemical properties of FeVO_4_ were synthesized by the hydrothermal method under different calcination temperatures (500 °C, 600 °C, and 700 °C) as an anode material for LIBs. The specific surface area (S_BET_) decreases while the crystallite size, particle size, and tap density increase with the calcination temperature. The morphologies of FV500, FV600, and FV700 also vary from nanorods to polygonal nanoparticles and microparticles, respectively. Furthermore, the specific capacities of FV500, FV600, and FV700 were reduced by increasing the calcination temperature and particle size. FV500 displays a higher specific capacity than FV600 and FV700 because of their large surface area and morphological nature. Moreover, the PAA binder, with its high mechanical and adhesive properties, strong hydrogen bonding, and nonfluorinated components, accommodates large volume changes during the cycling process and provides a stable cycle life. In contrast, PVDF mixed with FV500, FV600, and FV700 electrodes exhibited large capacity fading as a result of poor hydrogen bonding, weak van der Waals forces, and the presence of acidic species such as HF, LiF, and PF_5_, which further enhance the volume changes during the cycling process. Therefore, the calcination temperature determines high tap density, specific surface area (S_BET_), and the intrinsic nature of materials, and the appropriate choice of binder material for FeVO_4_ makes it an auspicious anode material for LIBs.

## Figures and Tables

**Table 1:** Summary of unit cell data and lattice parameters of FV500, FV600, and FV700, respectively.

FeVO_4_	Parameters			Δ Volume V = A°^3^	Crystallite Size (nm)	Lattice Strain (E) (%)
	a	b	c			
Standard	6.718	8.060	9.254	462.459	-	-
FV500	6.718	8.060	9.253	462.384	13.802	1.04
FV600	6.679	8.024	9.299	460.085	14.373	1.16
FV700	6.711	8.055	9.350	466.305	15.937	1.02

**Table 2:** Summary of the physicochemical properties of FV500, FV600, and FV700, respectively.

Temperature (°C)	Pore Volume (m^3^/g)	Pore Size (nm)	Tap Density (g/mL)	BET Area (m^2^/g)
FV500	3.124 × 10^-8^	20.819	0.105	60.030
FV600	4.681 × 10^-8^	5.472	0.218	34.222
FV700	3.049 × 10^-8^	4.955	1.057	24.616

## Data Availability

Not applicable.

## Supplementary Materials

The following supporting information can be downloaded at: https://www.mdpi.com/article/10.3390/ma16020565/s1.
