# Supplementary material for: Effect of Calcination Temperature on the Physicochemical Properties and Electrochemical Performance of FeVO4 as an Anode for Lithium-Ion Batteries
Source: Materials (Basel). 2023 Jan 6;16(2):565. doi: 10.3390/ma16020565 (PMC9866506; doi:10.3390/ma16020565)
Supplement: Supplementary file 1 [file materials-16-00565-s001.zip › materials-2090350-supplementary.pdf]

[Supporting Information]

Effect of Calcination Temperature on the Physicochemical and Electrochemical Performance of $FeVO_4$ as an Anode for LIBs

Faizan Ghani[1], Kunsik An[2], Dongjin Lee [1], *

[1]Department of Mechanical and Aerospace Engineering, Konkuk University, Seoul campus, 120 Neungdong-ro, Gwangjin-gu, 05029 Seoul, Republic of Korea

[2]Department of Mechatronics Engineering, Konkuk University, Glocal campus, 268 Chungwon-daero, Chungju-si, 27478, Republic of Korea

*Correspondence: Department of Mechanical and Aerospace Engineering, Konkuk University, Seoul campus, 120 Neungdong-ro, Gwangjin-gu, 05029 Seoul, Republic of Korea; djlee@konkuk.ac.kr; Tel.: +82-2-450-0452

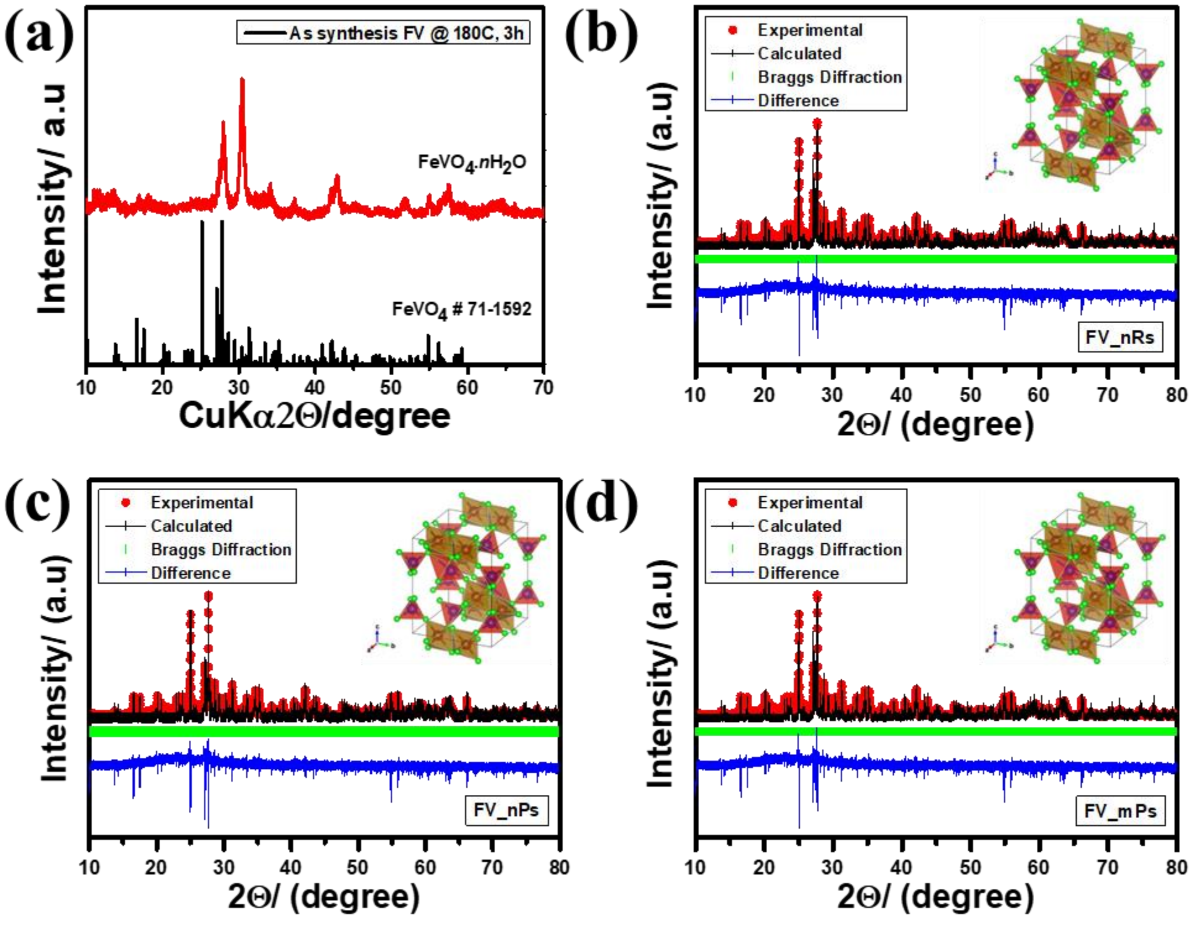

Figure S1. (a) XRD pattern of as synthesis $FeVO_4.nH_2O$ nanorods. Rietveld refinement of the XRD analysis of $FeVO_4$ at different temperature (b) 500°C, (c) 600°C, and (d) 700°C, respectively.

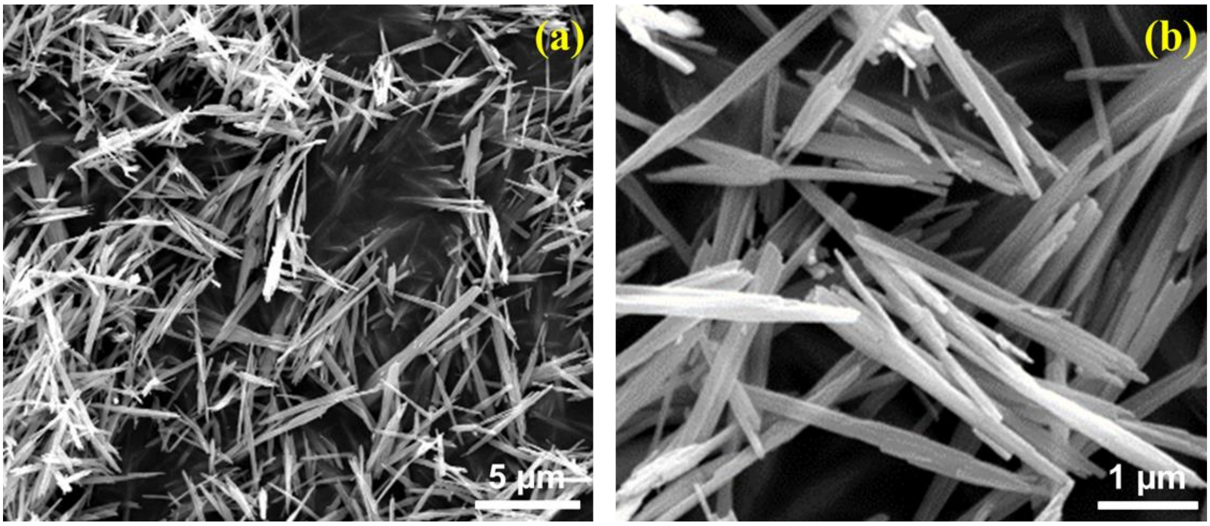

Figure S2. SEM images of as synthesis $FeVO_4.\ nH_2O$ before calcination at the magnification of (a) 5 µm and (b) 1 µm, respectively.

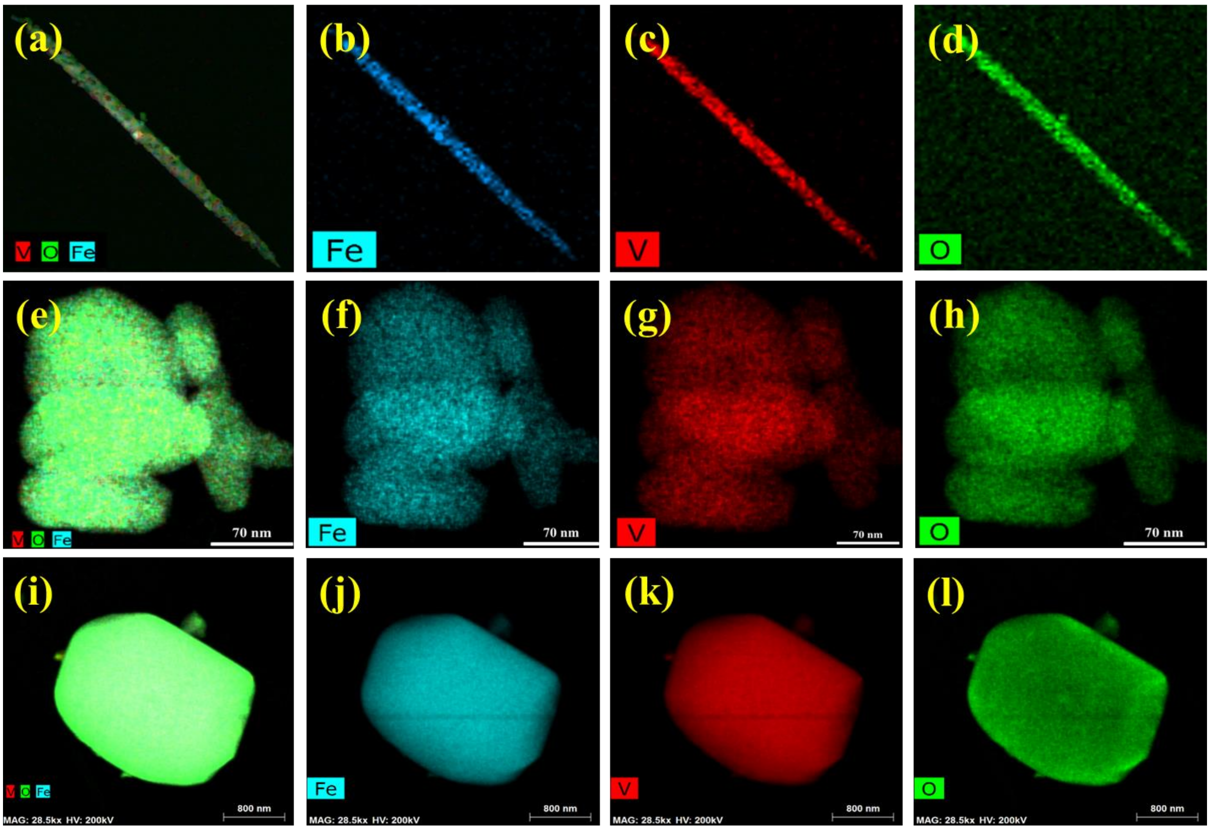

Figure S3. EDS-element mapping analysis of (a-d) FV500, (e-h) FV600, and (i-l) FV700, respectively.

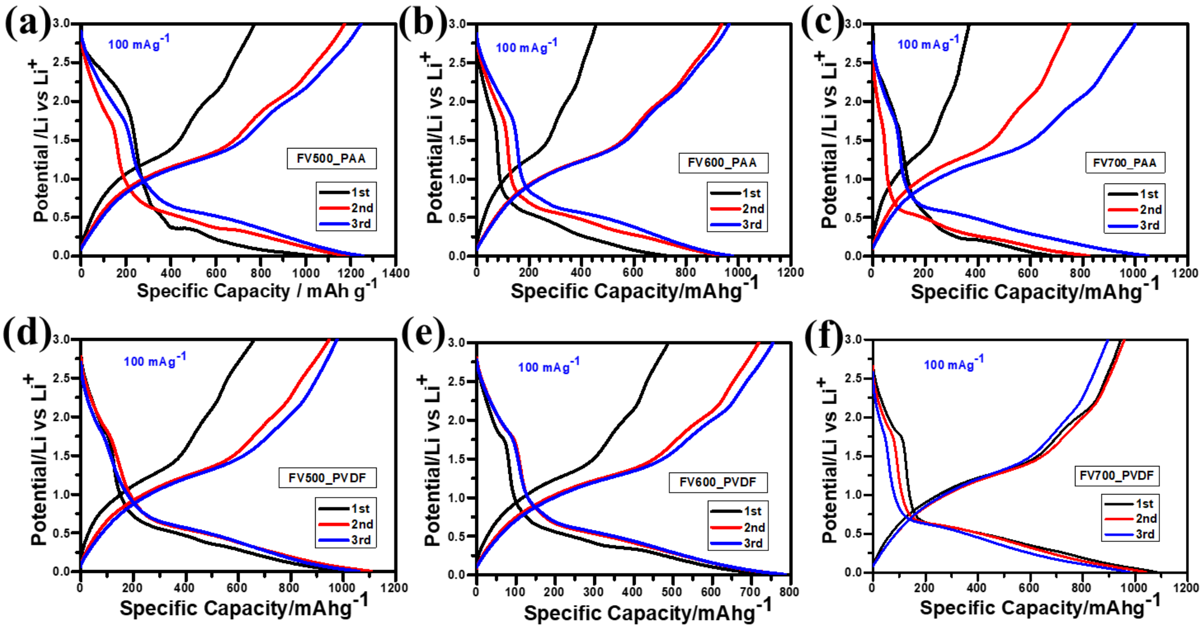

Figure S4. Galvanostatic discharge/charge profiles of (a, d) FV_nRs, (b, e) FV_nPs, and (c, f) FV_mPs fabricated with (a-c) 35 weights % PAA and (d-f) 5 weights % PVDF binders at the current densities of 100 mAg^{-1} within the voltage window of 3.0 V-0.005 V, respectively.
